# Structural Lability of *Barley Stripe Mosaic Virus* Virions

**DOI:** 10.1371/journal.pone.0060942

**Published:** 2013-04-17

**Authors:** Valentin V. Makarov, Eugeny V. Skurat, Pavel I. Semenyuk, Dmitry A. Abashkin, Natalya O. Kalinina, Alexsandr M. Arutyunyan, Andrey G. Solovyev, Eugeny N. Dobrov

**Affiliations:** 1 A. N. Belozersky Institute of Physico-Chemical Biology, Lomonosov Moscow State University, Moscow, Russia; 2 Biology Department, Lomonosov Moscow State University, Moscow, Russia; 3 Faculty of Bioengineering and Bioinformatics, Lomonosov Moscow State University, Moscow, Russia; University of Leeds, United Kingdom

## Abstract

Virions of *Barley stripe mosaic virus* (BSMV) were neglected for more than thirty years after their basic properties were determined. In this paper, the physicochemical characteristics of BSMV virions and virion-derived viral capsid protein (CP) were analyzed, namely, the absorption and intrinsic fluorescence spectra, circular dichroism spectra, differential scanning calorimetry curves, and size distributions by dynamic laser light scattering. The structural properties of BSMV virions proved to be intermediate between those of *Tobacco mosaic virus* (TMV), a well-characterized virus with rigid rod-shaped virions, and flexuous filamentous plant viruses. The BSMV virions were found to be considerably more labile than expected from their rod-like morphology and a distant sequence relation of the BSMV and TMV CPs. The circular dichroism spectra of BSMV CP subunits incorporated into the virions, but not subunits of free CP, demonstrated a significant proportion of beta-structure elements, which were proposed to be localized mostly in the protein regions exposed on the virion outer surface. These beta-structure elements likely formed during virion assembly can comprise the N- and C-terminal protein regions unstructured in the non-virion CP and can mediate inter-subunit interactions. Based on computer-assisted structure modeling, a model for BSMV CP subunit structural fold compliant with the available experimental data was proposed.

## Introduction

The architecture of viral capsids is largely based on either icosahedral or helical symmetry [1]. In recent years, spherical capsids with icosahedral symmetry were extensively studied by X-ray diffraction analysis and cryoelectron microscopy. By now, structures of many icosahedral viruses, which infect hosts belonging to all kingdoms of life, have been resolved at moderate or high resolution [2], [3], [4]. However, much less information has been obtained on the structure of helical viral capsids.

Capsids with the helical symmetry are typical for numerous rod-shaped and filamentous plant viruses. For many years, *Tobacco mosaic virus* (TMV), one of the best studied plant pathogens first identified more than one hundred years ago, was the only helical plant virus with resolved capsid structure. Conventional X-ray diffraction analysis of “20S disks” formed by the TMV coat protein (CP) carried out in the 1970s [5] and parallel studies of the whole TMV virions by X-ray fiber diffraction [6] made it possible to resolve the TMV structure at 4 Å resolution. Further progress in TMV structural analysis was made about three decades later when higher resolutions were achieved by both X-ray fiber diffraction studies (2.9 Å) [7] and cryoelectron microscopy (4.6 Å) [8], [9]. It should be noted, however, that such a resolution, even if some contradictions between the structures obtained by these two methods are not considered, is not sufficient to decipher the fine mechanisms of TMV self-assembly. The CP subunit of the TMV capsid comprises a single structural domain characterized by a bundle of four main α-helices. Two of the four main α-helices positioned radially in the plane perpendicular to the virion axis are called the ‘left radial helix’ (residues 111–135) and the ‘right radial helix’ (residues 73–86), while the two other main helices positioned at an angle to this plane are called the ‘left slewed helix’ (residues 19–33) and the ‘right slewed helix’ (residues 37–52). Additionally, the TMV CP structure includes two small α-helices located at the borders of the four-helix-bundle domain and a short β-strand of several residues. Both N- and C-terminal protein regions exposed on the outer surface of the TMV capsid [7]. The internal surface of the TMV virion, where adjacent subunits are packed very tightly, has a complex structure consisting of a series of H-bond stabilized reverse turns [7].

*Barley stripe mosaic virus* (BSMV, genus *Hordeivirus*) with rod-like helical virions [11] and the CP sequence distantly related to that of TMV [12] could be expected to be close to TMV in both the CP fold and the virion structure. However, studies of the CP role in viral infection suggest that the BSMV CP could be functionally different from the TMV CP. In particular, unlike the TMV CP, the BSMV CP is not required for virus long-distance transport through the phloem [13], implying that such transport involves non-virion forms of the viral genome.

In the last three decades, BSMV was the subject of detailed studies focused on the genome structure and expression, functions of encoded proteins in virus infection, and interaction with plant hosts [13]. On the other hand, the structure of the BSMV capsid received little attention in these years. Earlier electron microscopy of BSMV virions made it possible to determine that the virus capsid represents a protein helix about 22 nm in diameter with 24 protein subunits per turn of the helix and a pitch of 2.5–2.6 nm [11], [14], [15]. Although limited, these data clearly show that the structure of BSMV capsid differ from that of TMV, which is characterized by the CP helix 19 nm in diameter, 16.33 protein subunits per helical turn, and a pitch of 2.3 nm. In this context, structural studies of the BSMV virions and the BSMV CP may provide an insight into the molecular organization of helical viral capsids.

In this paper we used sensitive methods of protein analysis to determine a number of physicochemical characteristics of BSMV and its CP and the data obtained allowed us to propose a model of the BSMV CP structure.

## Materials and Methods

### BSMV propagation and isolation

The cloned infectious cDNAs of BSMV (strain ND18) [16] were subcloned under the control of the Act2 promoter and the NOS terminator, transferred into the agrobacterial vector pCambia, and used for transformation of *Agrobacterium tumefaciens* strain GV3101. Transformed agrobacteria were resuspended in agroinfiltration buffer (10 mM MES pH 5.6, 10 mM MgCl_2_, and 150 µm acetosyringone) and infiltrated into leaves of *Nicotiana benthamiana* plants. Leaves for virus isolation were harvested 2 weeks after agroinfiltration. The leaves were homogenized in a blender with three volumes of 0.5 M borate buffer pH 9.0. The mixture was incubated with 0.5% Triton X-100 for 20 min, extracted with ¼ volume of chloroform, and centrifuged at 10000 g for 20 min. The virus was precipitated from the supernatant by incubation with 4% PEG-6000 and 1.2% NaCl for 2 hours at room temperature. The mixture was centrifuged again at 10000 g, and the pellet was resuspended in 50 mM borate buffer pH 9. After clarification, the virus was pelleted by ultracentrifugation (2 hours at 100000 g) through a 1 ml cushion of 20% sucrose. The virus was resuspended in 5 mM phosphate buffer pH 7.5 and stored at +4°C. The BSMV yield was about 30 mg per 100 g of systemically infected leaves. The homogeneity of the resulting sample was evaluated by absorption spectroscopy, electron microscopy (Fig. S1A), and PAGE (Fig. S1D).

### Isolation of the BSMV CP

BSMV CP was isolated by LiCl treatment of BSMV virions [17]. Virus suspension was mixed with 1/3 volume of 13.6 M LiCl and incubated for 6 hours at −20°C. The virus RNA was precipitated by centrifugation for 15 min at 13000 g, and LiCl was removed against 5 mM phosphate pH 9.5 at +4°C to isolate the disks or by dialysis against 5 mM phosphate pH 7.0 followed by incubation at room temperature for 1–2 hours to isolate larger protein multimers. The disk and multimer formation was confirmed by electron microscopy (Sup Fig. S1B, C). The CP preparations were stored at +4°C.

### TMV and TMV CP propagation and isolation

TMV was purified by the previously described method [18]. 4S TMV CP was obtained by the acetic acid method [19]. TMV CP 20S disks were prepared by the 4S protein incubation in 0.05 M phosphate buffer pH 7.0 for 24 hours as described previously [40].

### Spectra measurements

Unless otherwise indicated, the phosphate buffer was used as the most suitable buffer for far-UV CD measurements. The absorption spectra in the 240–340 nm range were measured in cells with an optical path of 1 cm using a Hitachi UV-2600 spectrophotometer. The CD spectra were recorded in 1–2 mm (185–250 nm) cells at 25°C using a Chiroscan CD spectrometer (Applied Photophysics, UK). The spectra were measured in 5 mM phosphate buffer pH 7.5. The sample concentration was 100 μg/ml. The CD spectra were recorded at 0.5–1.0 nm/s with a baseline subtraction. The measured spectra were smoothed with the help of the instrument software. The values of [θ] were calculated taking the mean molecular weight of amino acid residues to be 110. The intrinsic fluorescence spectra were recorded in 1 cm cells using a FluoroMax spectrofluorometer (HORIBA Jobin Yvon, USA) at 25°C. Fluorescence of protein at the concentration of 0.03 mg/ml was excited in 1 mM phosphate buffer pH 7.5 at 280 nm, and the emission spectra were recorded in the 300–400 nm range.

### Structure modeling

For homology modeling of the BSMV CP structure, a multiple sequence alignment of CPs encoded by rod-shaped viruses of the family *Virgaviridae* was generated using ClustalW2 [20]. The homology modeling was performed with the MODELLER 9.7 software (www.salilab.org/modeller/) using the crystal structure of the CP CGMMV (pdb ID: 1CGM). The interactions between adjacent subunits were not taken into account in the model.

### Differential scanning calorimetry

DSC experiments were performed on a DASM-4 microcalorimeter (Biopribor, Russia) with capillary platinum 0.47-mL cells at a scanning rate of 1°C/min. The second heating was used as the instrument baseline because of the irreversible denaturation observed for the investigated sample. The chemical baseline was calculated and subtracted using the Origin 1.16 software.

### Dynamic light scattering

The sizes of BSMV virion and CP were determined using dynamic light scattering (DLS). All experiments were carried out on a ZetaSizer NanoZS instrument (Malvern, England) with the laser wavelength of 633 nm. The data were analyzed using the Dispersion Technology Software version 5.10. The samples (at about 100 μg/ml) were measured in 10 mM phosphate buffer. BSMV virions were disrupted with 0.1% SDS.

## Results

### Optical characteristics of native and disassembled BSMV virions

**Figure 1:**
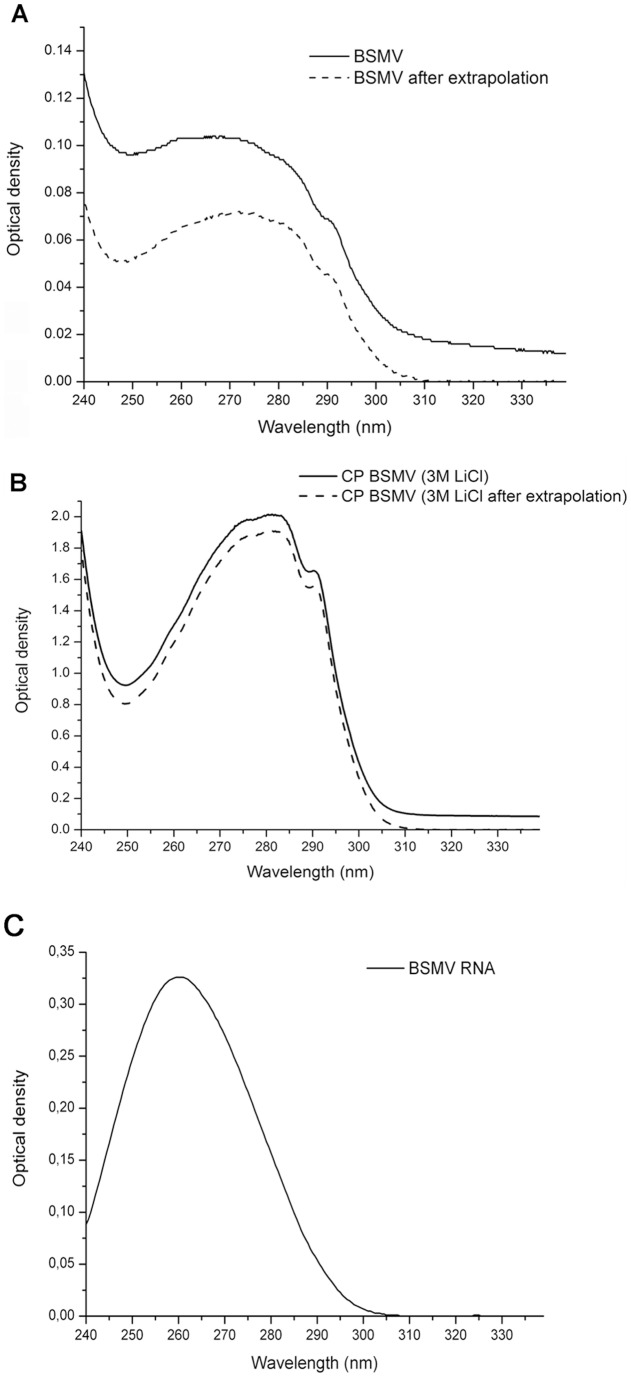
UV absorption spectra of intact BSMV virions (A) and of LiCl prepared isolated BSMV. Both samples are in 5 mM PB pH 7.5. Directly measured and scattering-corrected spectra are shown. Light-scattering correction was performed with extrapolation procedure as described in Tikchonenko et al. 1966 [41].

Preparations of BSMV (strain ND18) isolated from *Nicotiana benthamiana* plants were used to measure the UV spectra of BSMV virions (Fig. 1A) and BSMV CP (Fig. 1B). The BSMV CP (198 amino acids) was found to have a high absorption coefficient (E_0.1%,280_  = 1.60) due to five Trp and six Tyr residues in its sequence. For BSMV virions we used absorbtion coefficient (E_0.1%,260_) equal to 2.4. The BSMV CP absorption spectrum was measured in 3M LiCl solution immediately after disassembly of the virions and RNA precipitation, which occurs under these conditions [11], was typical for proteins of plant virus, showing that the protein was efficiently separated from the viral RNA. Actually, the E_260_/(E _280_ ratio equals 0.68 for CP BSMV after RNA precipitation, which corresponds to the pure RNA-free protein. Such BSMV CP preparations were characterized by a low turbidity (Fig. 1B), thus confirming previous observations that the BSMV CP was unable to form multimers under these conditions [21].

**Figure 2:**
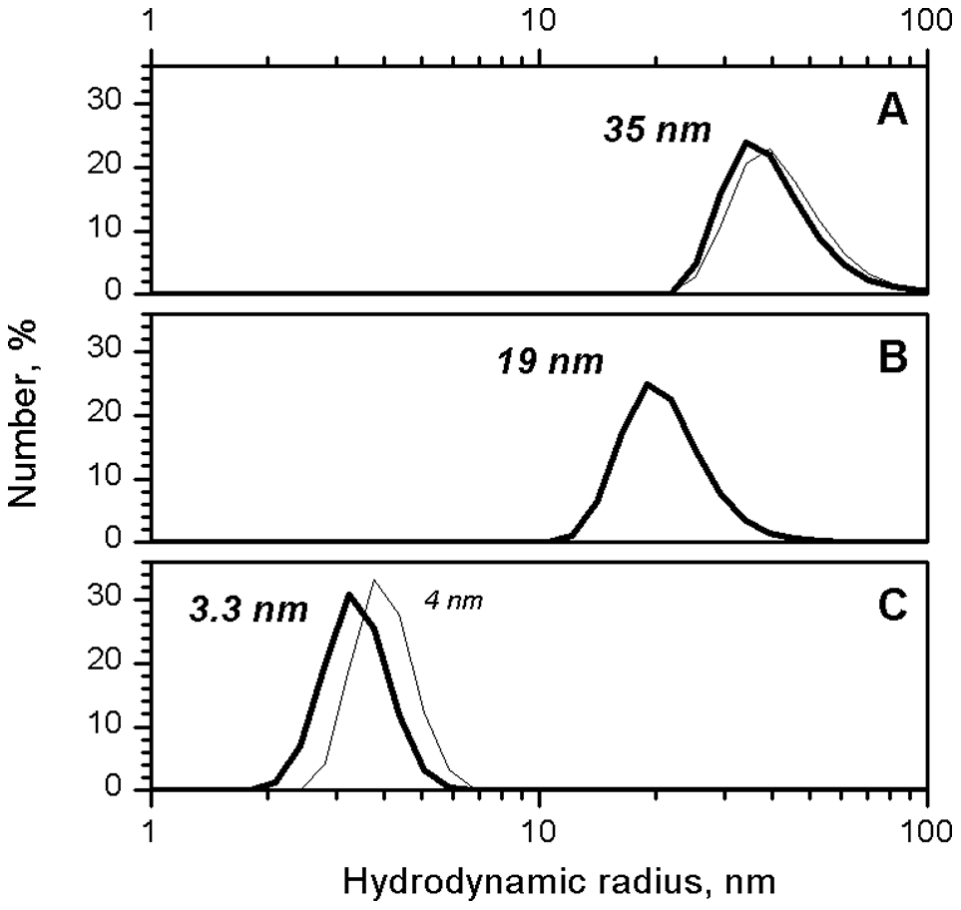
DLS size distribution for native BSMV virions (A), multimeric BSMV CP (B), and disaggregated BSMV CP in 0.1% SDS (C). TMV virions and 4S CP are shown as thin lines in A and C).

Dynamic laser light scattering (DLS), the method making possible to reveal the size distribution of small particles dispersed in solution [22], was used to determine the SDS concentration sufficient to cause the disassembly of BSMV virions. The BSMV virions were found to be completely disassembled in 0.1% SDS at 25°C (Fig. 2). TMV strain U1 from the Collection of the Department of Virology, Moscow State University, was used as the control. Note that TMV CP 4S is larger than BSMV CP, which further confirms that BSMV virions disassemble to mono- or dimers under these conditions. The TMV virions were previously shown to be disassembled in 0.1% SDS only when the temperature was increased to 60°C [23], while virions of filamentous plant viruses were disassembled in conditions similar to those found for BSMV [24].

**Figure 3:**
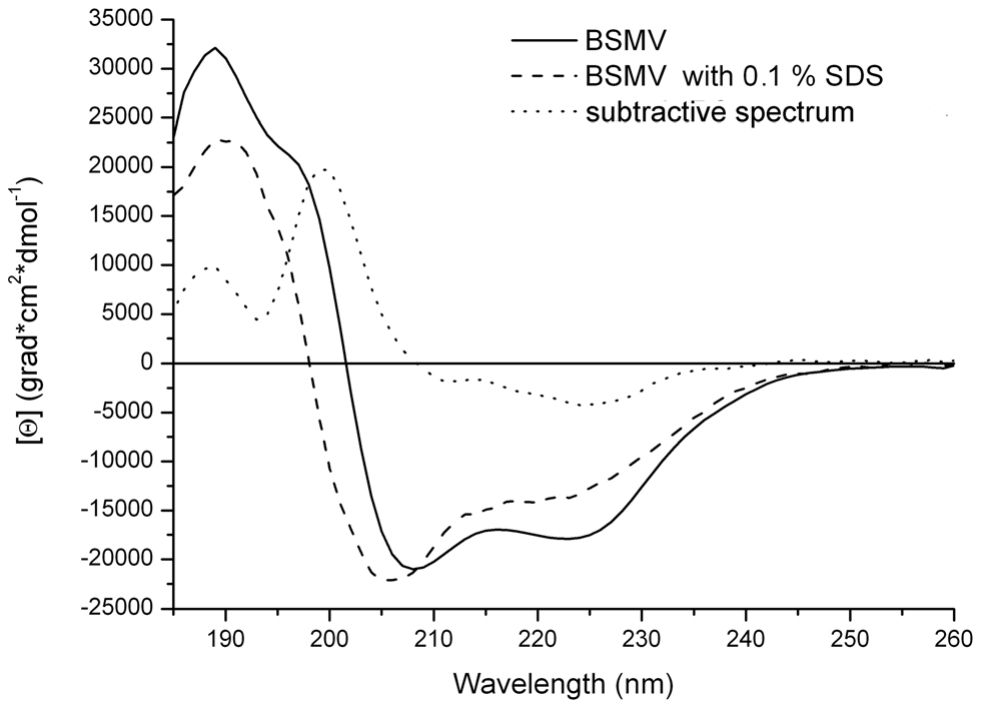
Far UV CD spectra of intact 0.1% SDS-disrupted BSMB virions and their subtractive spectrum (intact minus disrupted spectrum) are presented. Spectra in 5 mM PB were measured in 1 mm cells at 25°C.

Further, the circular dichroism (CD) spectroscopy was used to compare the structural properties of BSMV virions and the non-virion BSMV CP. The CD spectrum of BSMV virions in the far UV region (190–250 nm) had no unusual peaks and was typical for proteins with high content of α-helices (Fig. 3). The BSMV far-UV spectrum differed from those of a number of flexuous filamentous viruses by a more pronounced minimum at 218 nm (with the molar ellipticity in this peak being 0.85 that at 208 nm) and the lack of an abnormal peak at 228 nm, which was found for *Potato virus X* (PVX) [25]. The spectrum of intact BSMV in the 185–200 nm region had the principal maximum of 32000 grad*cm^2^*dmol^−1^ at 189 nm. This peak was approximately 30% higher than the peak at 208 nm and had a shoulder at 197 nm with the intensity of 58% of the 189 nm peak (Fig. 3). Analysis of the BSMV CD spectrum using the web-based service K2D2, which estimates protein secondary structure from CD data [26], predicted 38% α-helices and 12% β-structure elements in BSMV CP incorporated into virions, while the Dichroprot software [27] predicted 48% α-helices and 24% β-structures; however, both estimates seem excessive, particularly, for β-structures (Table 1).

**Table 1:** Structural component composition of CP BSMV aggregation forms from far-UV CD spectra (as predicted by Dichroprot).

Sample Name	α (%)	β (%)	Unordered elements (%)
BSMV virions	48	24	28
Disrupted BSMV virions	42	17	41
BSMV CP monomer	36	17	47
BSMV CP disks	16	32	51
BSMV CP aggregates	12	37	52

The CD spectra of the BSMV CP prepared by the treatment of the virions with 0.1% SDS at 25°C demonstrated that the virion disassembly resulted in a slight shift of the main minimum from 208 to 206 nm while the molar ellipticity of the peak remained almost unchanged, and a considerable signal decrease in the region of 210–240 nm (Fig. 3). The strong signal in this region is mostly attributed to the presence of β-structures [28]. Therefore, a much higher [θ]_218_ value observed for the intact BSMV virions than for the non-virion CP obtained upon virion disassembly suggested the existence of a significant fraction of β-structures in the intact virions. The [θ]_218_/[θ]_208_ ratio decreased from 0.85 to 0.60 upon the BSMV virion disruption. Additionally, the spectrum of non-virion CP exhibited a shift of the zero point from 201 to 198 nm, a considerable decrease (down to 23000 grad*cm^2^*dmol^−1^) of the main maximum at 189 nm, and the absence of the shoulder at 197 nm. All these changes in the CD spectrum of the BSMV CP could be explained by the loss of a considerable fraction of α- and β-structure elements and the corresponding increase in the proportion of unstructured elements upon the disassembly of BSMV virions in 0.1% SDS (Table 1).

**Figure 4:**
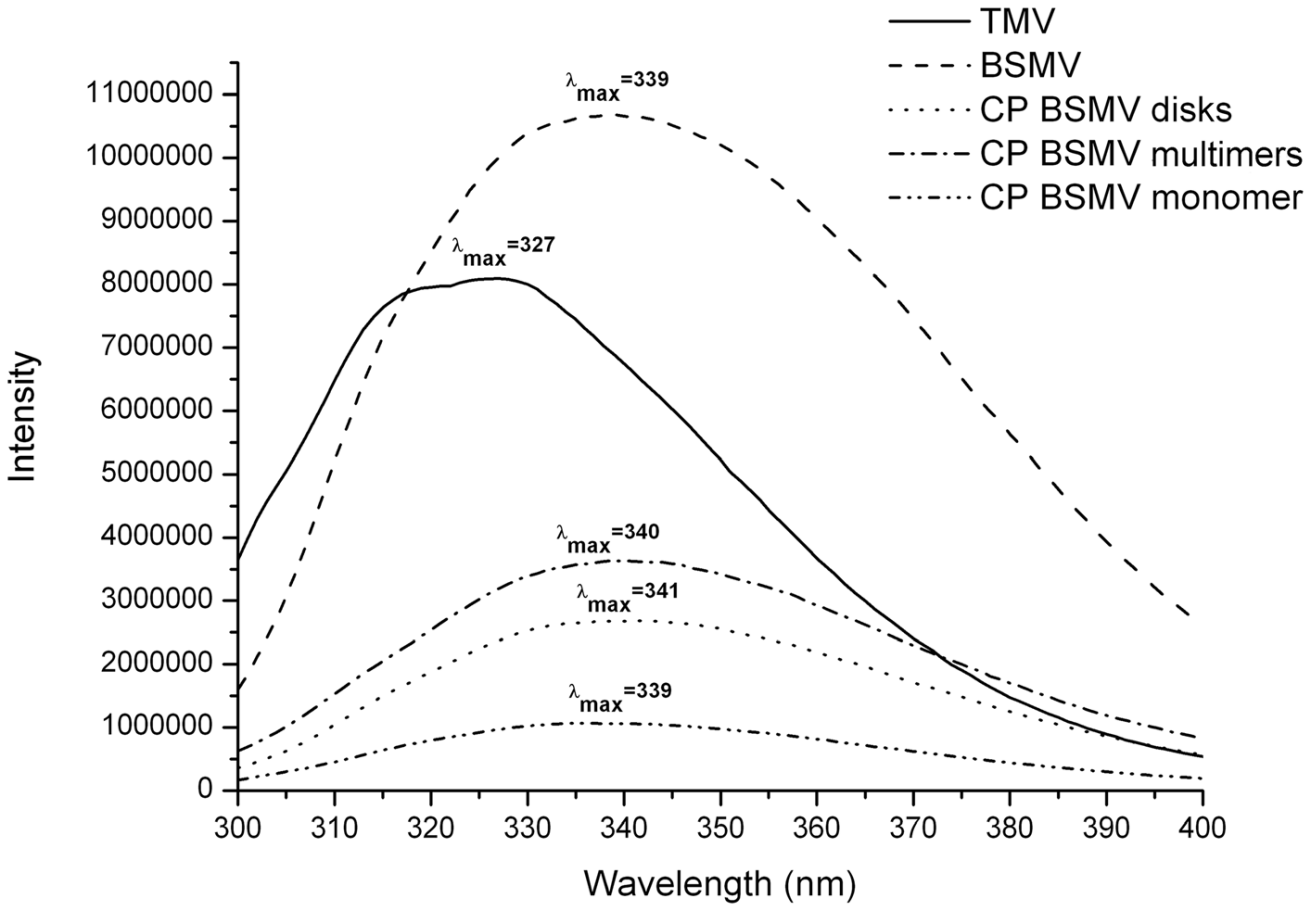
Intrinsic fluorescence spectra of BPSV CP incorporated into the virions, LiCl-isolated monomeric CP, and several CP multimeric forms. Excitation by 280 nm light at 25°C. Samples concentration was about 30 mkg\ml.

Moreover, the intrinsic fluorescence of BSMV CP either incorporated into virions or existing in solution in a number of multimeric forms was measured upon excitation with 280 nm light. For comparison, the spectrum of the intact virions of TMV, whose CP contains approximately the same percentage of Trp and Tyr residues as the BSMV CP, was measured. Aromatic amino acids in the TMV CP, both incorporated into virions and in the non-virion form, are known to reside in a hydrophobic environment; therefore the spectra of their fluorescence have a maximum at approximately 327 nm [29]. The intrinsic fluorescence of the BSMV CP incorporated into virions appeared to be even higher than that of TMV virions in the same conditions (Fig. 4), suggesting a pronounced tertiary structure in the BSMV CP molecules. The BSMV fluorescence maximum was located at 339 nm, which indicated that the Trp residues in the BSMV CP could reside in a more hydrophilic environment than in the TMV CP. However, due to the large number of aromatic residues in the BSMV CP, the details of their environment could not be inferred from the intrinsic fluorescence spectra.

### Optical characteristics of different multimeric forms of BSMV CP

The spectra of intrinsic fluorescence were measured for different non-virion forms of the BSMV CP, namely, the monomeric protein, preparations of the CP disks, and preparation of the higher multimeric protein. In 3M LiCl, the position of the spectrum maximum remained unchanged, while the fluorescence intensity decreased 10 times (Fig. 4). This observation suggested that the environment of aromatic residues in the monomeric CP became disordered, while the formation of CP disks and their further association partially restored the structured environment of these residues inherent to the BSMV CP incorporated into virions.

**Figure 5:**
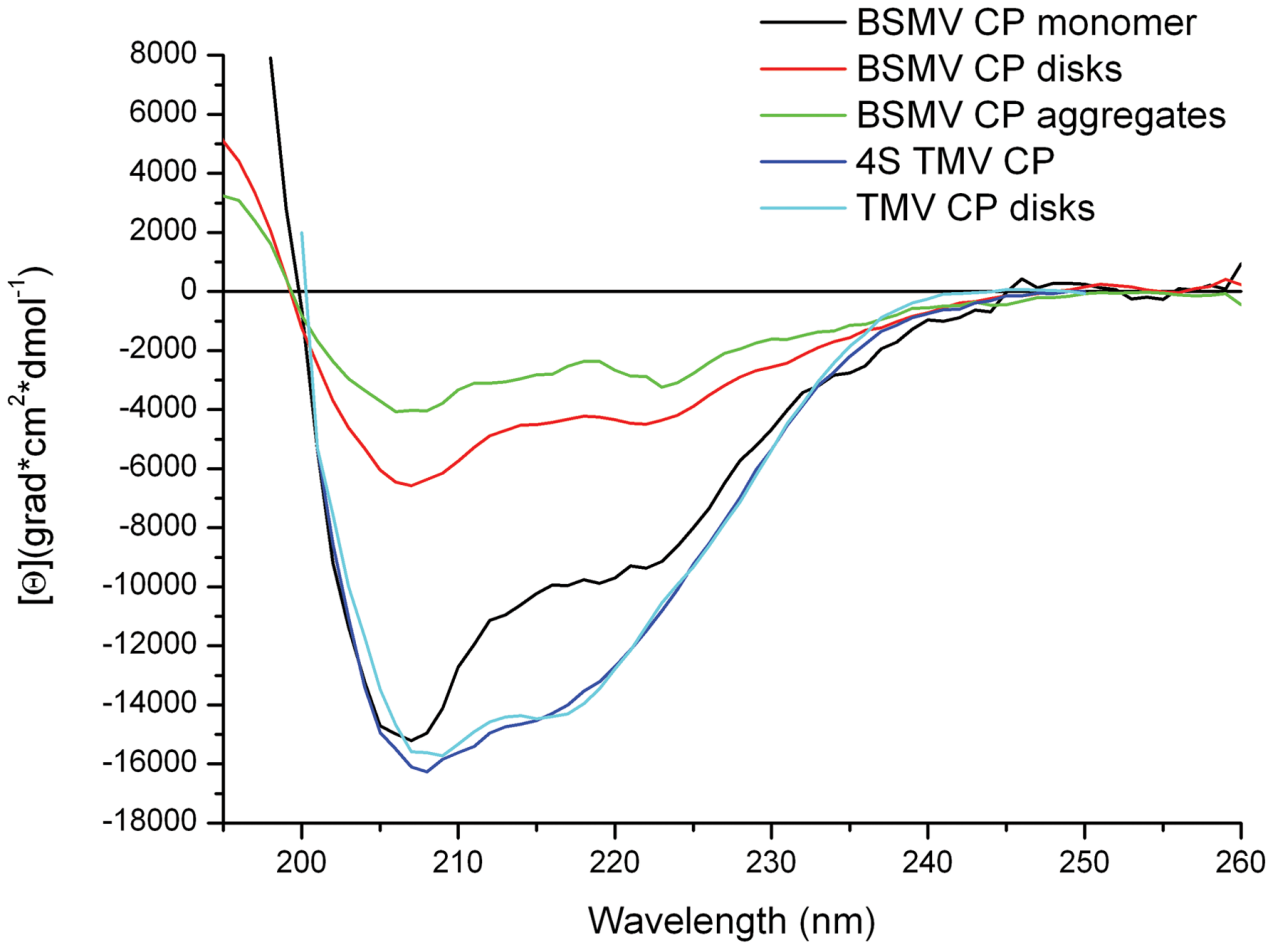
Far-UV CD spectra of different multimeric forms of BSMV CP. Monomeric CP spectrum was measured in 3M LiCl; other forms, in 5 mM PB.

The CD spectra in the far UV region were recorded for different multimeric forms of the non-virion BSMV CP. We were able to measure, although only up to 200 nm, the CD spectrum for the BSMV CP in 3M LiCl solution. This spectrum was typical for many proteins with a [θ]_208_ value of −15000 grad*cm^2^*dmol^−1^ (Fig. 5), which corresponded to 21% α-helices and 26% β-structure elements according to the K2D2 estimation. Additionally, preparations of BSMV CP in 3M LiCl were subjected to dialysis against the 5 mM phosphate buffer at pH 7.0 or pH 9.0 and used for CD spectra measurements at 25°C. The 5 mM phosphate buffer pH 9.0 was very close to the conditions described previously for the formation of the CP disks from the monomeric CP [21]. It was found that in this buffer the ellipticity minimum at 208 nm considerably reduced compared to the protein preparation in 3M LiCl (Fig. 5), which corresponded to a decrease in the proportion of α-helices in the protein structure. At the same time, the [θ]_218_/[θ]_208_ ratio increased from 0.6 to 0.7.

As demonstrated previously, the incubation of the BSMV CP in the 5 mM phosphate buffer pH 7.0 for 1–2 hours at 25°C converted the disks into higher order multimers stable upon further incubation at 4°C [21]. The CD spectra in the far UV region recorded for the BSMV CP in these conditions demonstrated a further, compared to pH 7.0, reduction the ellipticity minimum at 208 nm from −6500 to −4500 grad*cm^2^*dmol^−1^ (Fig. 5). The [θ]_218_/[θ]_208_ ratio, known to reflect the proportion between β-structure elements and α-helices in protein structure, increased to 0.75 to approach that of the intact BSMV virions, while the ellipticity maximum shifted from 192 nm, the value corresponding to α-helices, to 188 nm, the value specific for β-structure elements. The increased proportion of β-structure elements with the multimerization of the BSMV CP is corroborated by the deconvolution of the spectra using Dichroprot (Table 1). Notice that that transition from 4S CP to the disks in the case of TMV was not accompanied by β-structure formation, and both spectra were typical for proteins containing ∼50% α-helices in their structure (Fig. 5).

### Differential scanning calorimetry

**Figure 6:**
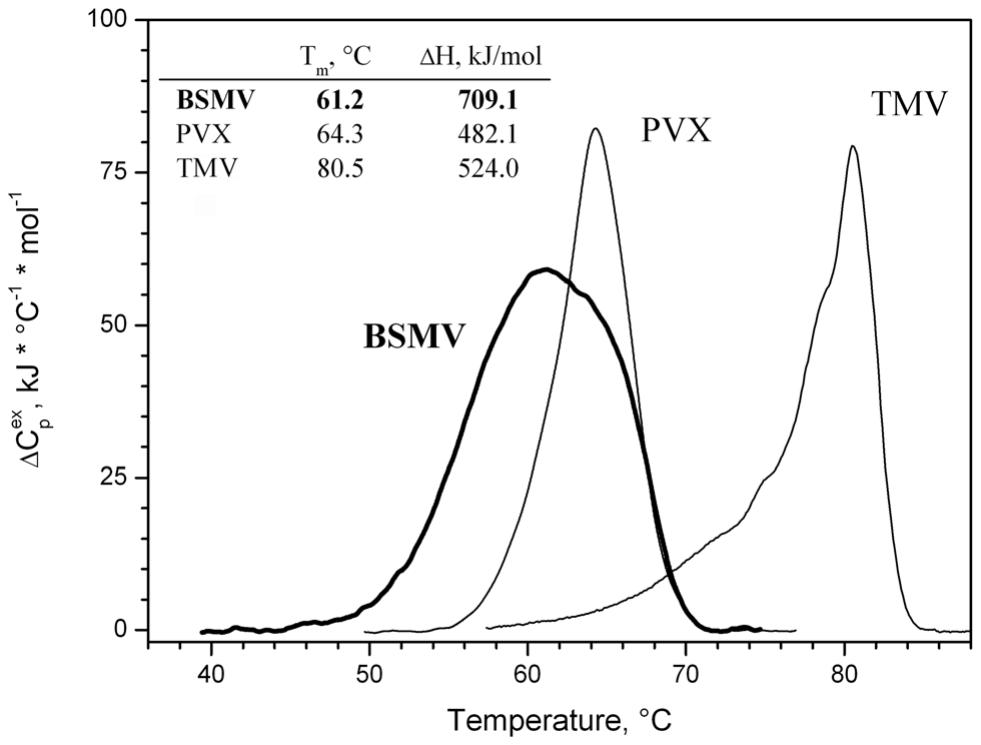
DSC melting curves for BSMV CP in 5 mM PB, rod-like TMV virions, and flexuous potato virus X virions.

The structure of BSMV virions was further analyzed by their heat denaturation using differential scanning calorimetry (DSC) [30]. The DSC curves obtained for the BSMV virions had single thermal peaks with melting temperatures of 61°C and ΔH of 700 kJ/mole (Fig. 6). These data show that the BSMV CP consists of a single structural domain. The melting curve for BSMV virions was measured at pH 9.0, since at lower pH these virions precipitated even after a brief heating [11]. The DSC Tm value for BSMV virions proved to be somewhat lower compared not only to TMV, but also to flexuous PVX (Fig. 6) [24]. This observation suggested that at least in some aspects the BSMV virions could be even less stable then the virions of some flexuous plant viruses.

### Homology modeling of the BSMV CP

We tried to model the BSMV CP subunit structure based on the resolved structures of tobamovirus CPs. A multiple sequence alignment of CPs encoded by rod-shaped viruses of the family *Virgaviridae* was generated using ClustalW2 [20]. It was corrected in accordance with the previously published sequence analysis [12] and the comparisons of the BSMV CP secondary structure predicted by the PSIPRED algorithm (PMID: 10869041) to the secondary structure elements of proteins with known fold (Fig. S2). On the basis of the alignment, the CP of *Cucumber green mottle mosaic virus* (CGMMV) was identified as the protein exhibiting the closest sequence relation to the BSMV CP among viral CPs with resolved structure (20% identity, 31% similarity). The CGMMV CP structure has been resolved at 3.4 Å [31].

**Figure 7:**
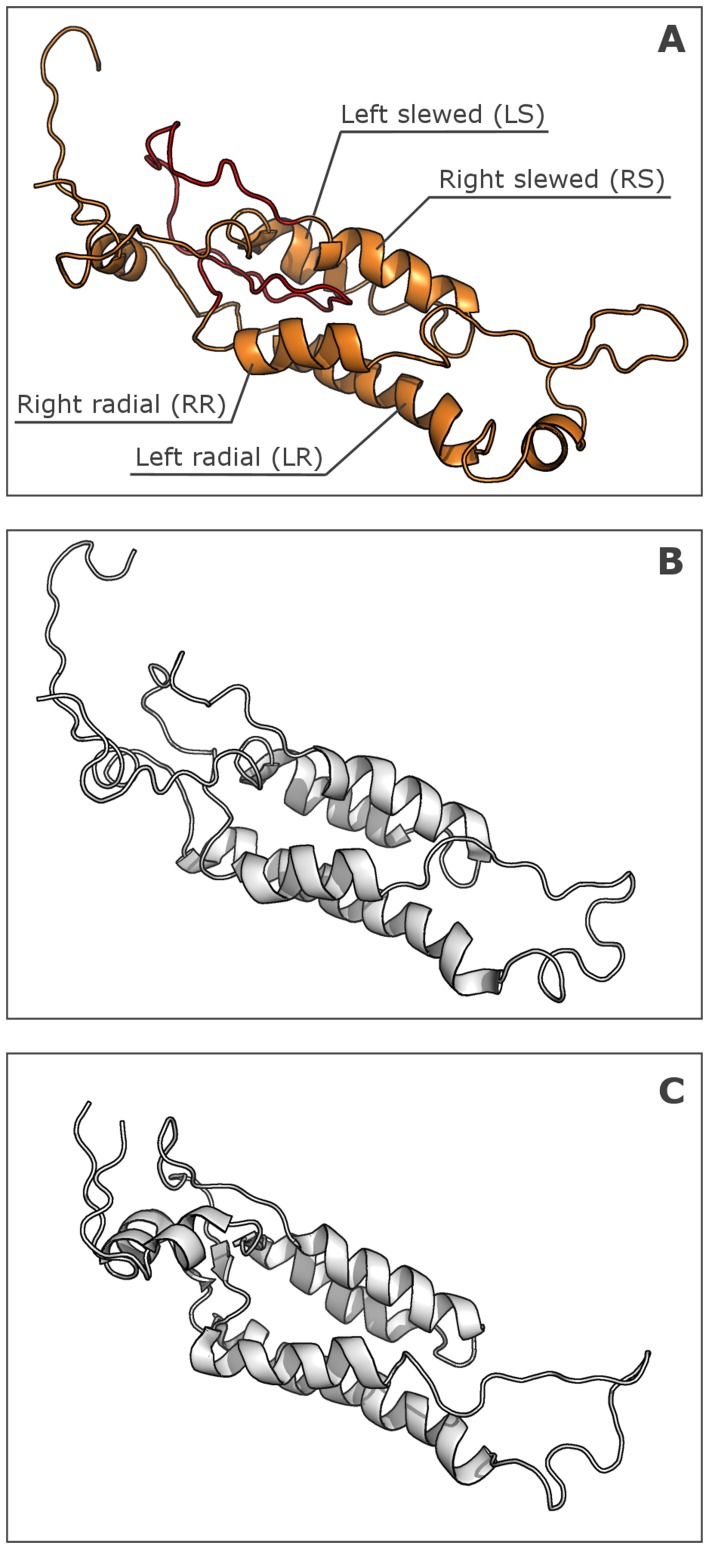
Homology-derived model of BSMV CP (A) and PDB models of CGMMV(B) and U1 TMV CPs (C). Virion axis is on the right.

Using the CGMMV CP structure as a template, we were able to build a structure model for most of the BSMV CP sequence, namely the region from residue 13 to the protein C-terminus (residue 198) (Fig. 7 and Fig. S3). The structure of the 12 N-terminal residues in the BSMV CP could not be modeled, and this region was consistently found to be unfolded in all our predictions. Consistent with our expectations, the protein loops and the C-terminus, being larger in the BSMV CP compared to the TMV CP, were not well aligned to the known CGMMV structure and, on the other hand, had different conformations in five best BSMV CP models (not shown); therefore, their precise location awaits experimental resolution.

According to the proposed model, the BSMV CP subunit was characterized, as expected, by many features common with the CGMMV CP and also some significant differences. As in the CGMMV CP, the core of BSMV CP molecule was represented by a four-helix bundle (Fig. 7). As in the CGMMV CP, the four α -helices in the BSMV CP include two radial and two slewed helices, and their lengths are similar to those of the respective helices in the CGMMV CP. On the other hand, the amino acid sequence of the BSMV CP is considerably longer than that of the CGMMV CP, and the “excessive” residues of the BSMV CP are most probably located in unstructured regions in the protein termini and in loops between the helices. Both N- and C-termini of the protein were located on one molecule side corresponding to the outer surface of the helical virion, while the protein part presumably located in the virion central channel formed H-bond-stabilized reverse turns. On the other hand, the loop between α-helix-2 and α-helix-3 (Fig. 7), which could be located at the periphery of the BSMV virion, proved to be 33 amino acids long, which was much larger than in the CGMMV. The CP of PapMV was recently reported to possess a somewhat shorter unstructured CP region (residues 126–135) most probably located on the virion outer surface [10]. The content of α-helices in the proposed model was 35%, which was in a good agreement with calculations based on the BSMV CD spectrum (38% α-helices, see above). The data on the Trp intrinsic fluorescence also agreed with the model: experimental data suggested a hydrophilic environment for the Trp residues, thus excluding their location in the molecule hydrophobic core. According to the model, most of the Trp residues were located in the N- and C-terminal regions located on the surface of BSMV virion. Additionally, the size of the modeled BSMV CP molecule was 6 nm, which agreed with the hydrodynamic diameter determined by the DLS analysis.

## Discussion

Formation of ribonucleoprotein complexes (RNPs) comprising viral genomic RNA and virus-encoded proteins is essential for many steps in plant virus life cycle, such as protection of viral genome from attacks by cellular enzymes, transport of viral RNA cell-to-cell and long distance through the phloem, and interaction with different virus-encoded and cell partner proteins. The BSMV genomic RNA, in addition to virion RNPs, can be incorporated into a specialized transport RNP formed by the movement protein TGBp1 [13], [32]. These two types of BSMV RNPs differ both structurally and functionally: the CP-formed virion RNPs represent stable tightly packed structures primarily aimed at the maximum protection of viral genome, while the TGBp1-formed transport RNPs, being more flexible [33], are destined for the transport through plasmodesmal microchannels, which implies a structural lability of the latter RNPs including their conditional structural remodeling [34]. Similarly, the TMV cell-to-cell movement requires a viral movement protein forming transport-competent RNPs [35]. In filamentous plant viruses such as potexviruses, specialized transport RNPs are not formed, and the virions perform both protective and transport functions [36], [37], [38]. Consequently, flexuous virions of such viruses are more labile and less resistant to external factors [24], [39]a. Thus, virions of helical plant viruses can differ considerably in their lability: most stable and rigid virions are typical for viruses having a transport RNP formed by dedicated movement proteins, while most labile virions are found in viruses where the virion itself can serve as a transport RNP.

Based on general considerations, the structural features of rod-shaped BSMV virions and BSMV CP should be similar to those of TMV. A moderate degree of sequence similarity between the BSMV and TMV CPs [12] seem to support this prediction. However, in this paper we report that the BSMV virions are considerably less stable that the TMV virions and close in their lability to virions of filamentous plant viruses. Moreover, the lability of the BSMV CP was found to depend on the degree of protein multimerization: the intrinsic protein fluorescence data revealed that the monomeric CP had a high lability, the CP-formed disks were less labile, and the CP incorporated into virions was rather stable. These observations suggest that the CP structure can change upon homologous interactions. Additionally, the intrinsic protein fluorescence spectra suggested that the Trp residues in the BSMV CP could be located in much more hydrophilic environment than the TMV CP Trp residues residing in the molecule hydrophobic core. These data are in agreement with the BSMV CP model proposed in this paper: according to the model, most of the Trp residues are situated in the protein regions located on the outer virion surface and therefore are not involved in the formation of the α-helical protein core.

The proposed BSMV CP model differs from the TMV CP structure in its long unstructured N-terminal sequence and the virion surface-located loop between α-helix-2 and α-helix-3 (residues 68–100). A somewhat shorter similar structure is described for the CP of PapMV, which has flexuous filamentous helical virions [10]; it is tempting therefore to speculate that these protein regions are at least in part responsible for a higher lability of the BSMV virions compared to TMV lacking such structure elements in its CP.

Based on the CD spectra of the BSMV virions and the BSMV CP, we propose that, in contrast to TMV and other plant viruses with known CP structure, the CP subunits of the BSMV virion contain a significant proportion of β-structure elements. We presume that β-structure regions, which are found by CD spectroscopy in the BSMV CP incorporated in native virions but not in non-virion monomeric CP, can be formed during virion assembly involving the CP polymerization. These β-structure regions may include the N- and C-terminal protein regions unstructured in the non-virion CP, as well as the loop between α-helix-2 and α-helix-3 located at the virion periphery. We propose that a transition from the unstructured state to the β-structure may be induced by protein-protein and protein-RNA interactions arising during the capsid self-assembly. It would be important to know whether the CP forms intramolecular β-structures or cross-β-structures involving regions of the neighboring CP subunits. The latter possibility would be reminiscent of the interaction of PapMV capsid subunits [10], which draws a structural parallel between BSMV and flexuous filamentous viruses. A distant structural resemblance of BSMV to filamentous viruses is in agreement with the fact that the BSMV virions appeared to be less stable than the TMV virions and are comparable to those of flexuous helical plant viruses in their stability. One can hypothesize that the increased lability of rigid rod-shaped BSMV virions could be due to the unusual properties of the BSMV CP N- and C- terminal regions, namely, (i) their strong conservation among hordeiviruses, which strikingly contrast other helical plant viruses exhibiting a high degree of sequence variability of the CP terminal regions, and (ii) the presence of five invariant Trp residues in the N-terminal region of hordeivirus CPs. It is possible that the extended extra-loop is required for the BSMV virions to interact with different CP-coated segments of viral genomic RNAs, thus increasing the infection efficiency and ensuring that all three genomic RNAs enter the cell. The above-mentioned high capacity of BSMV CP for oligomerization indirectly confirms this assumption. We presume that the BSMV virion lability reported in this paper might account for some unstudied biological properties of hordeiviruses.

## Supporting Information

Figure S1 **The homogeneity of the samples BSMV virions (A, D), disks (B) and aggregates (C) was evaluated by electron microscopy (A, B, C) and PAGE (D).**
(TIF)

Figure S2 **Amino acid sequence alignment of the CPs encoded by different rod-shaped viruses.** BSMV, *Barley stripe mosaic virus* (genus *Hordeivirus*); PSLV, *Poa semilatent virus* (genus *Hordeivirus*); LRSV, *Lychnis ringspot virus* (genus *Hordeivirus*); CGMMV, *Cucumber green mottle mosaic virus* (genus *Tobamovirus*); TMV, *Tobacco mosaic virus* (genus *Tobamovirus*); RMV, *Ribgrass mosaic virus* (genus *Tobamovirus*); HLSV, *Hibiscus latent Singapore virus* (genus *Tobamovirus*); TRV, *Tobacco rattle virus* (genus *Tobravirus*); and PEBV, *Pea early browning virus* (genus *Tobravirus*).
(TIF)

Figure S3 **3D struture of CP BSMV in the PDB file.**
(PDB)
