# Supplementary figures and images for: Structural Lability of Barley Stripe Mosaic Virus Virions
Source: PLoS One. 2013 Apr 17;8(4):e60942. doi: 10.1371/journal.pone.0060942 (PMC3629216; doi:10.1371/journal.pone.0060942)

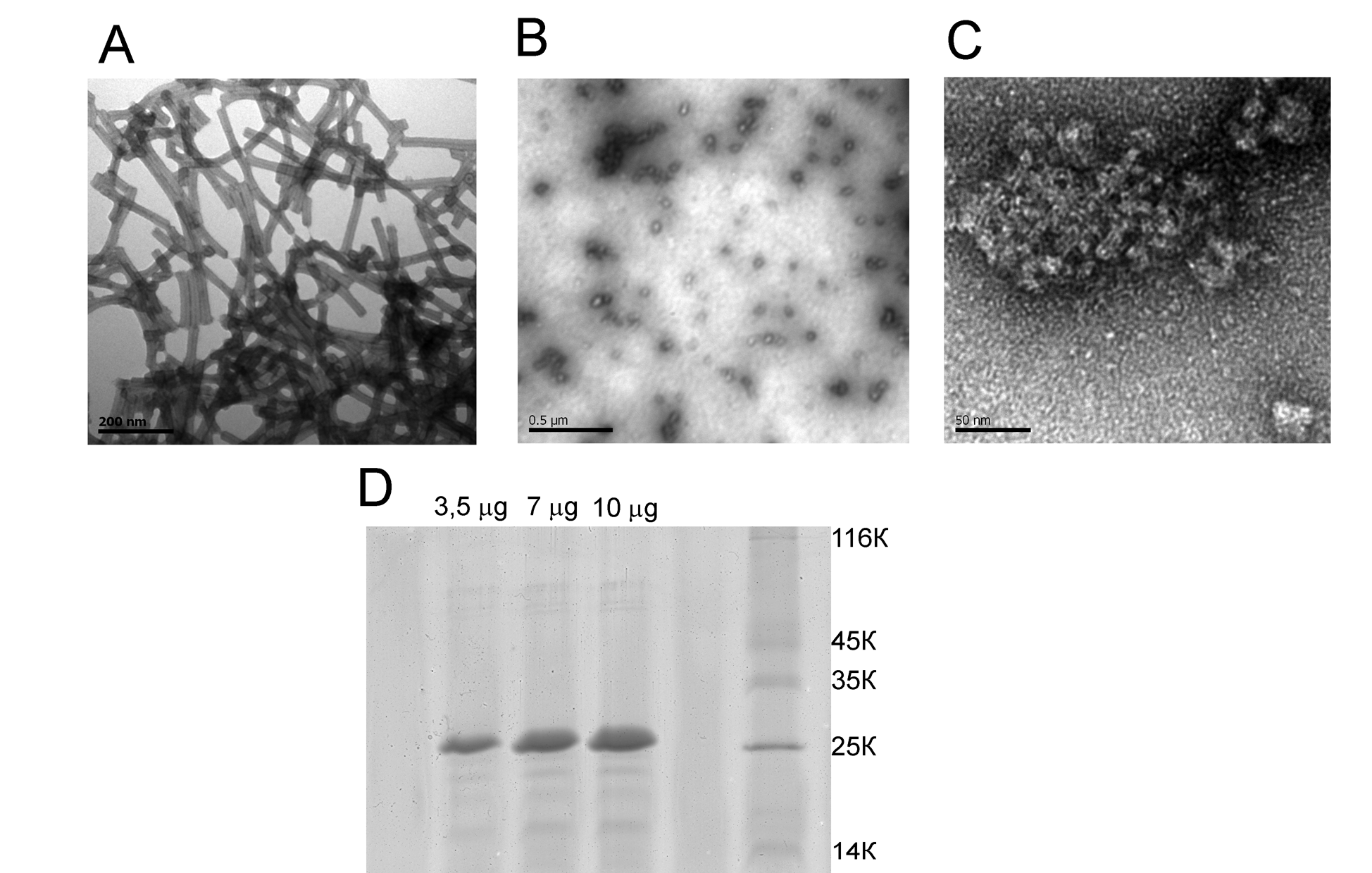

Supplement: Figure S1 — The homogeneity of the samples BSMV virions (A, D), disks (B) and aggregates (C) was evaluated by electron microscopy (A, B, C) and PAGE (D). (TIF) [file pone.0060942.s001.tif]

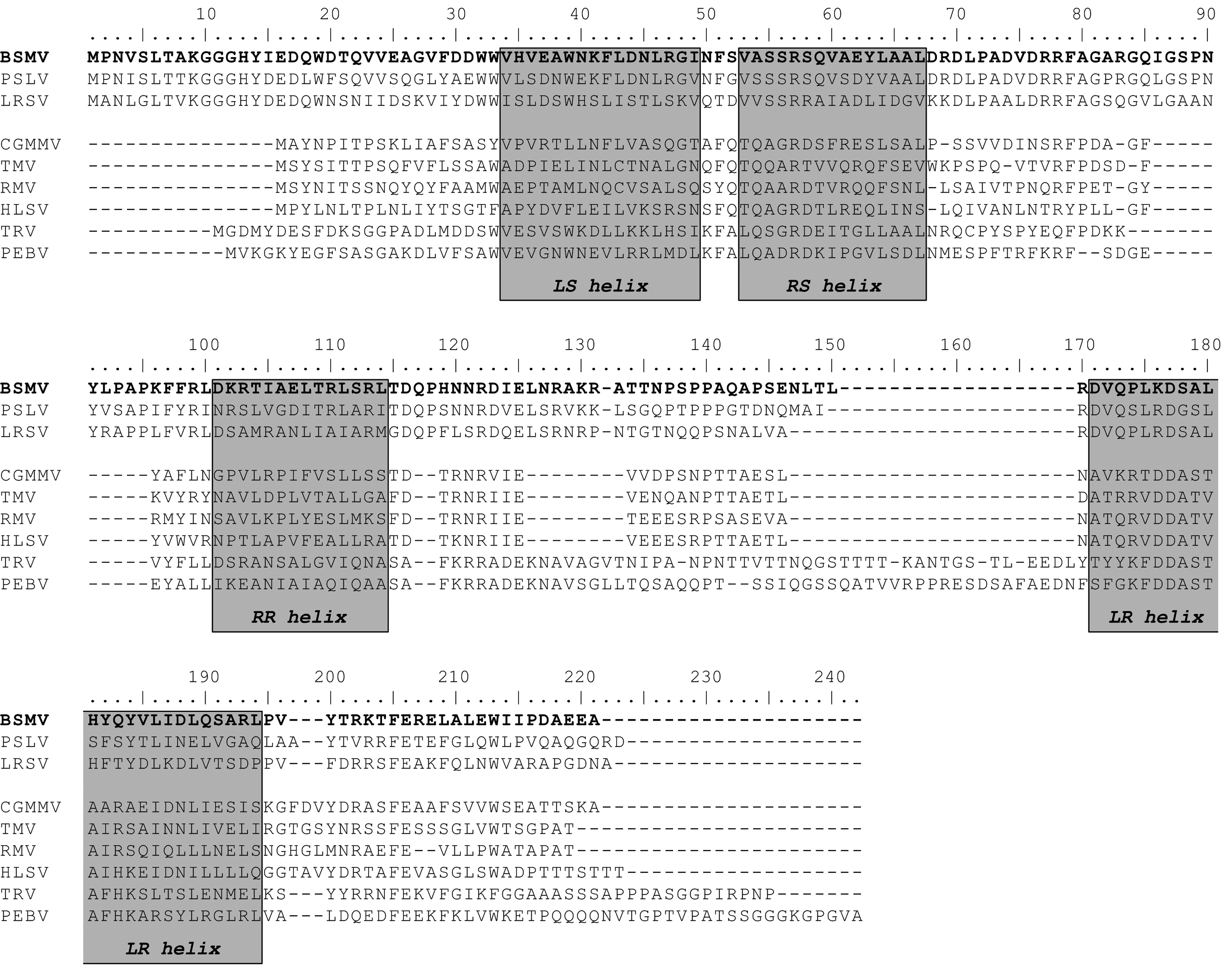

Supplement: Figure S2 — Amino acid sequence alignment of the CPs encoded by different rod-shaped viruses. BSMV, Barley stripe mosaic virus (genus Hordeivirus); PSLV, Poa semilatent virus (genus Hordeivirus); LRSV, Lychnis ringspot virus (genus Hordeivirus); CGMMV, Cucumber green mottle mosaic virus (genus Tobamovirus); TMV, Tobacco mosaic virus (genus Tobamovirus); RMV, Ribgrass mosaic virus (genus Tobamovirus); HLSV, Hibiscus latent Singapore virus (genus Tobamovirus); TRV, Tobacco rattle virus (genus Tobravirus); and PEBV, Pea early browning virus (genus Tobravirus). (TIF) [file pone.0060942.s002.tif]
